# Transient visual evoked potential abnormalities in ADNP syndrome

**DOI:** 10.1186/s11689-026-09688-y

**Published:** 2026-04-01

**Authors:** Tess Levy, Hailey Silver, Nurit Benrey, Abigail Siegel, Christina Layton, Vance Zemon, James Gordon, Joseph D. Buxbaum, Alexander Kolevzon, Paige M. Siper

**Affiliations:** 1 Seaver Autism Center for Research and Treatment, Icahn School of Medicine at Mount Sinai, New York, NY 10029 USA; 2 Department of Psychiatry, Icahn School of Medicine at Mount Sinai, New York, NY 10029 USA; 3 Ferkauf Graduate School of Psychology, Yeshiva University, Bronx, NY 10461 USA; 4 Department of Psychology, Hunter College, City University of New York, New York, NY 10065 USA; 5 Friedman Brain Institute, Icahn School of Medicine at Mount Sinai, New York, NY 10029 USA; 6 Department of Genetics and Genomic Sciences, Icahn School of Medicine at Mount Sinai, New York, NY 10029 USA; 7 Department of Neuroscience, Icahn School of Medicine at Mount Sinai, New York, NY 10029 USA; 8 The Mindich Child Health and Development Institute, Icahn School of Medicine at Mount Sinai, New York, NY 10029 USA; 9 Department of Pediatrics, Icahn School of Medicine at Mount Sinai, New York, NY 10029 USA

**Keywords:** ADNP syndrome, Helsmoortel-van der Aa syndrome, Visual evoked potentials, Neurodevelopmental disorder

## Abstract

**Background:**

ADNP syndrome is a rare genetic disorder associated with global developmental delay/intellectual disability, autism, aberrant behavior, and medical comorbidities. Sensory symptoms represent a core clinical feature, even in those without autism spectrum disorder (ASD). Differences in visual evoked potentials (VEPs), an objective measure of excitatory and inhibitory postsynaptic activity, have been reported in other genetic neurodevelopmental disorders and have yet to be examined in ADNP syndrome.

**Methods:**

Transient VEPs (tVEP) were collected from 12 children with ADNP syndrome, 46 autistic children without a known genetic cause, and 19 typically developing children. Time- and frequency-domain variables were compared between groups.

**Results:**

Significant differences were found between the ADNP and TD groups in amplitude (P_60_-N_75_, N_75_-P_100_), latency (P_60_, N_75_), and magnitude-squared coherence (MSC). Significant differences were also found between the ADNP and ASD group in latency (P_60_, N_75_) and MSC (Band 2, 14–28 Hz).

**Conclusions:**

VEP abnormalities in children with ADNP syndrome compared to an ASD group and controls were identified. Weaker amplitudes in the ADNP group are consistent with prior research in other genetic neurodevelopmental syndromes. Longer latencies and diminished 14–28 Hz band activity, however, are distinct findings and represent an important area of continued study to explore the presence of syndrome-specific VEP profiles. Establishing VEP biomarkers for ADNP syndrome is a critical direction for future clinical trials in the syndrome.

## Background

ADNP syndrome is a genetic neurodevelopmental disorder caused by pathogenic variants in the *ADNP* (activity dependent neuroprotective protein) gene (OMIM: 615873). *ADNP* is involved in chromatic remodeling and transcriptional regulation [1]. Individuals with ADNP syndrome present with global developmental delays, intellectual disability, and autism or autism traits [2–9]. Sensory differences are a common feature of ADNP syndrome, specifically sensory seeking behaviors, which are present regardless of autism status [9, 10]. Medically, individuals with ADNP syndrome can present with a wide array of features affecting neurological, ocular, cardiac, gastrointestinal, dental, endocrine, and urogenital systems [2–9].

Visual evoked potentials (VEPs) are an objective measure of excitatory and inhibitory postsynaptic activity elicited in response to specific visual stimuli and VEP differences have been reported in ADNP syndrome mouse models [11]. Transient VEPs (tVEPs) are of particular interest as contrast-reversing checkerboard stimuli produce a well-characterized waveform that examines the functional integrity of visual pathways in humans [12, 13]. Waveform peaks, or positive deflections, and troughs, or negative deflections, represent the sum of excitatory and inhibitory postsynaptic activity. P_60_, the initial positive peak, represents activation of the primary visual cortex from the lateral geniculate nucleus (LGN), typically occurs at ~ 60 ms and reflects inhibitory activity. N_75_, the first negative trough, represents excitatory postsynaptic activity occurring at ~ 75 ms, and P_100_, the second positive peak, represents inhibitory activity occurring at ~ 100 ms. In the time domain, peak-to-trough amplitudes measure the strength of responses and latency represents the timing of each peak or trough.

tVEP abnormalities have been described in other genetic disorders including Phelan-McDermid syndrome (PMS) [14], Rett syndrome [15, 16], Fragile X syndrome [17], FOXG1 syndrome [16], MECP2 Duplication syndrome [16], and CDKL5 deficiency disorder [16]. Importantly, characterizing these abnormalities have led to tVEPs being used as an outcome measure in interventional clinical trials (e.g., NCT06662188, NCT05187377). Here, we assessed tVEPs in children with ADNP syndrome compared to autistic children without a known genetic cause and typically developing (TD) controls.

## Methods

### Participants

Participants were enrolled in three groups: ADNP, ASD, and TD. Participants were between the ages of 2–12. The ADNP group included 12 children with a genetically confirmed diagnosis of ADNP syndrome. Usable data was unable to be obtained from two additional ADNP participants due to inability to fixate on the screen for the duration of the 60-s run. The ADNP group harbored variants in *ADNP*, that were classified as likely pathogenic or pathogenic by a CLIA-approved laboratory and confirmed by the team’s genetic counselor (TL). The ASD group consisted of 46 autistic children. Autism diagnoses were established using the Autism Diagnostic Observation Schedule – 2nd edition (ADOS-2), developmental history, and expert clinical judgement to confirm participants all met Diagnostic and Statistical Manual of Mental Disorders, 5th Edition (DSM-5) criteria for ASD. The TD group consisted of 19 children with no known developmental or psychiatric disorders based on caregiver report. Cognitive testing was completed for all participants using a developmentally appropriate assessment. One participant (ADNP group) was medicated for seizures. Table 1 displays demographic information for each cohort. This study was approved by the Mount Sinai Program for the Protection of Human Subjects.

**Table 1:** Summary of Demographics

	ADNP (*n* = 12)	ASD (*n* = 46)	TD (*n* = 19)	*p*
Age	8.56 (3.20)	7.58 (2.86)	7.03 (2.91)	0.367
Sex (Male, Female)	7, 5	43, 3	5, 14	< .001
FSIQ/DQ	36.90 (14.51)	55.30 (31.71)	111.94 (13.18)	< .001

Descriptives for the study sample. Age and full-scale IQ/DQ (FSIQ/DQ) are quantified by *M* (*SD*). One-way ANOVA analyses were used to examine group differences for age and full-scale IQ. Group differences for sex were examined with chi-square analyses. Full scale IQ data were missing from 1 TD child and 3 ASD participants

### VEP administration & stimulus conditions

Stimulus presentation, data collection, and preliminary electroencephalographic (EEG) pre-processing were completed with a VEP system (EvokeDx (*n* = 73); Neucodia (*n* = 4 ADNP). Neucodia is the precursor to EvokeDx with compatible pre-processing. A standard 60-second contrast-reversing 32 × 32 high-contrast checkerboard stimulus was presented on EvokeDx (17 × 17 deg field) and on Neucodia (10 × 10 degree field) with a 1 Hz square-wave temporal modulation, and one second of adaptation prior to the 60-second run [13]. This testing protocol followed International Society for Clinical Electrophysiology of Vision (ISCEV) recommendations [12]. Participants all had a caregiver and two research coordinators present during testing. One research coordinator was responsible for the technical aspects of VEP data collection, and a second coordinator was responsible for behavior management to ensure participants were looking at the screen and comfortable for the duration of data collection. In addition, VEP systems included infrared eye tracking to objectively monitor gaze fixation, and a noise/outlier detection feature to alert the examiner to movement or poor visual fixation. The standard transient VEP condition was administered to 16 children with ADNP, 12 of whom provided usable data included in these analyses.

### VEP pre-processing & analysis

A discrete Fourier transform (DFT) was applied to extract the harmonic frequency components (1 through 100 Hz) of the tVEP responses. The contrast-reversing checkerboard conditions produce symmetrical responses and therefore odd harmonics were not included in the analyses. The waveform was filtered post data collection removing any components over 48 Hz (which includes the 60 Hz component that represents a large amount of line noise). The remaining even harmonics were then used to reconstruct the waveforms. Amplitude and phase measures were calculated using cosine and sine coefficients of each frequency component.

**Fig. 1:**
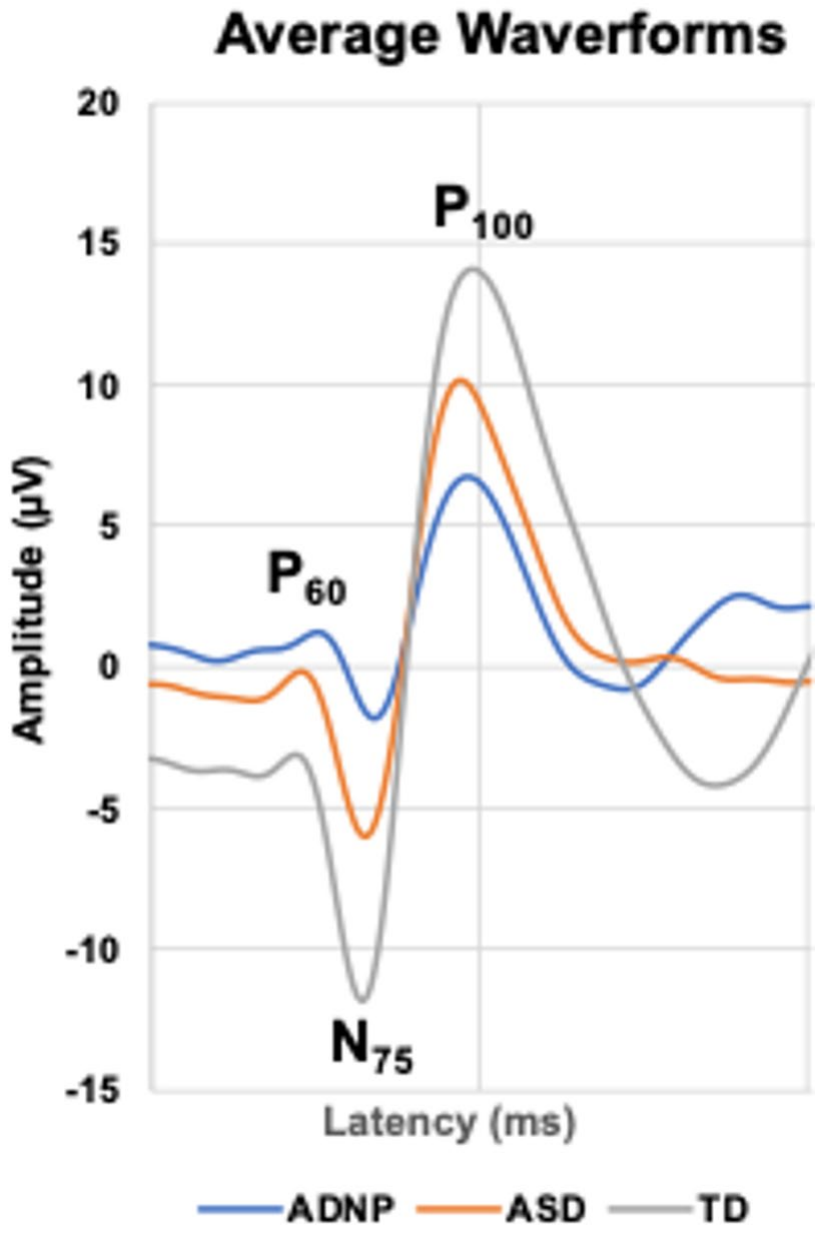
Average VEP Waveforms. Legend: Average waveforms for the TD, ASD, and ADNP groups

Per ISCEV standards [12], each individual waveform was visually inspected and P_60_, N_75_, P_100_ amplitudes (µV) measured from peak-to-trough and associated peak time (ms) were selected. In addition to these time-domain measures, frequency-domain variables included magnitude-squared coherence (MSC) calculations to measure the integrity of the entire response within specific frequency bands: Band 1 (6–12 Hz), Band 2 (14–28 Hz), Band 3 (30–40 Hz), and Band 4 (42–48 Hz). An MSC of 1 indicates all signal and no noise, while a signal of all noise produces an MSC value of ~ 0.1. Higher MSC values indicate stronger activity within that frequency band synchronized to the stimulus Fig. 1.

### Statistical analyses

Multiple regression analyses were used to determine group differences for tVEP variables while accounting for age. Sex was explored as a covariate given differences between groups but was not a significant term and therefore not retained in the models. Age was a significant covariate in the model and was therefore retained. Statistics and figures were created in RStudio.

## Results

### Time-domain

#### Amplitude

**Fig. 2:**
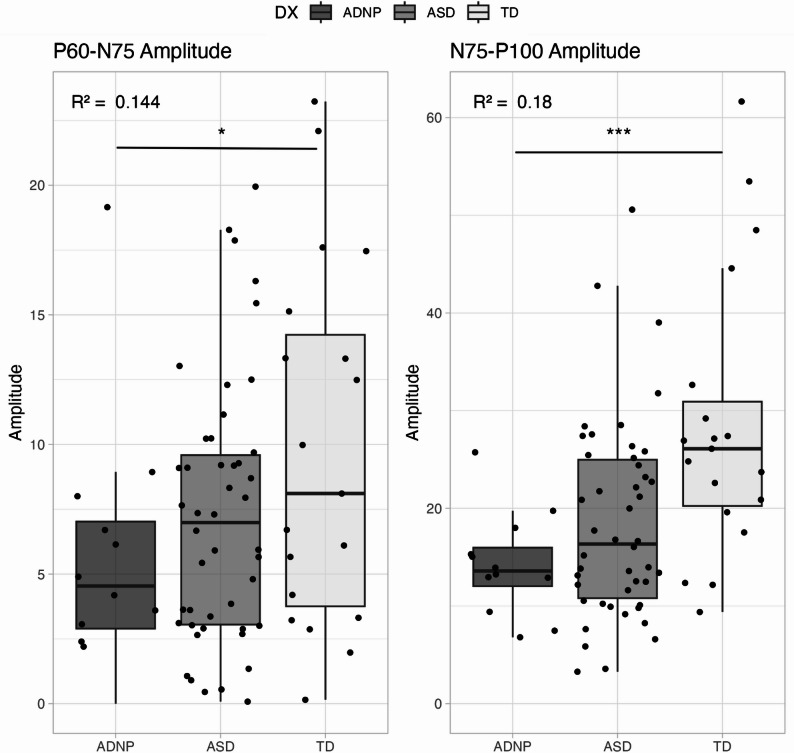
Amplitude Variables. Legend: Boxplots showing amplitude differences between P_60_-N_75_ (left) and N_75_-P_100_ (right). Colors represent the different groups: dark grey – ADNP, medium grey – ASD, and light grey – TD. Asterisks represent significance levels: * < 0.05, ** < 0.01, *** < 0.001; *p* values reflect group differences from the regression model. R^2^ value located in the top left-hand corner

P_60_-N_75_ amplitude was smallest for the ADNP group, and significantly smaller compared to the TD group (Table 2; Fig. 2). Similarly, N_75_-P_100_ amplitude was significantly smaller in the ADNP group compared to the TD group. The ASD group fell in between the two groups.

**Table 2:** Group Means (Standard Deviations) and Pairwise Comparisons for VEP and MSC Variables

Variable	ADNP	ASD	TD	ADNP vs. ASD	ADNP vs. TD
P_60_-N_75_ Amplitude	5.77 (4.9)	7.25 (5.1)	9.84 (7.0)	2.0 (1.7) *p*=.246	4.9 (2.0) *p*=.015
N_75_-P_100_ Amplitude	14.21 (5.3)	18.46 (10.1)	28.45 (14.2)	4.4 (3.5) *p*=.221	14.4 (4.0) *p*=.0007
P_60_ Latency	53.16 (11.1)	47.25 (5.1)	45.49 (5.6)	-6.4 (2.1) *p*=.003	-8.5 (2.4) *p*=.0006
N_75_ Latency	69.92 (8.7)	65.35 (6.2)	64.48 (3.6)	-4.6 (2.0) *p*=.028	-5.4 (2.3) *p*=.022
P_100_ Latency	98.25 (10.4)	95.10 (6.3)	100.33 (9.7)	-3.3 (2.6) *p*=.207	1.83 (3.0) *p*=.54
MSC Band 1 (6–12 Hz)	0.38 (0.1)	0.36 (0.2)	0.51 (0.2)	-0.0 (0.05) *p*=.89	0.15 (0.1) *p*=.014
MSC Band 2 (14–28 Hz)	0.29 (0.2)	0.41 (0.2)	0.50 (0.2)	0.1 (0.1) *p*=.018	0.2 (0.1) *p*=.0004
MSC Band 3 (30–40 Hz)	0.19 (0.1)	0.20 (0.1)	0.29 (0.2)	0.0 (0.0) *p*=.576	0.1 (0.1) *p*=.023
MSC Band 4 (42–48 Hz)	0.14 (0.1)	0.13 (0.1)	0.14 (0.1)	-0.0 (0.0) *p*=.94	0.0 (0.0) *p*=.87

*Legend*: Mean and standard deviation of each VEP variable by group. Coefficients, standard errors, and *p* values for comparisons between ADNP and ASD and ADNP and TD groups for each VEP variable. Values are presented as mean (standard deviation). Pairwise comparisons reflect mean differences (SE) with associated *p*-values. *ADNP* ADNP syndrome group, *ASD* autism spectrum disorder group, *TD * typically developing group, *MSC* magnitude-squared coherence

#### Latency

**Fig. 3:**
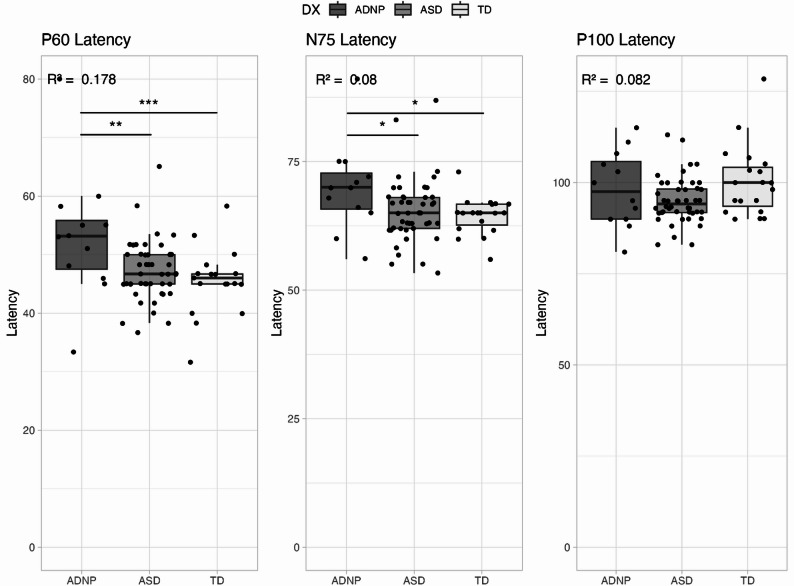
Latency Variables. Legend: Boxplots showing latency at P_60_ (left), N_75_ (middle), and P_100_ (right). Colors represent the different groups: dark grey – ADNP, medium grey – ASD, and light grey – TD. Asterisks represent significance levels: * <0.05, ** <0.01, ***<0.001; *p* values reflect group differences from the regression model. R^2^ value located in the top left-hand corner

Latency at P_60_ and N_75_ was significantly longer in the ADNP group compared to both ASD and TD groups (Table 2; Fig. 3). P_100_ latency did not differ between groups.

### Frequency domain

#### Magnitude squared coherence

**Fig. 4:**
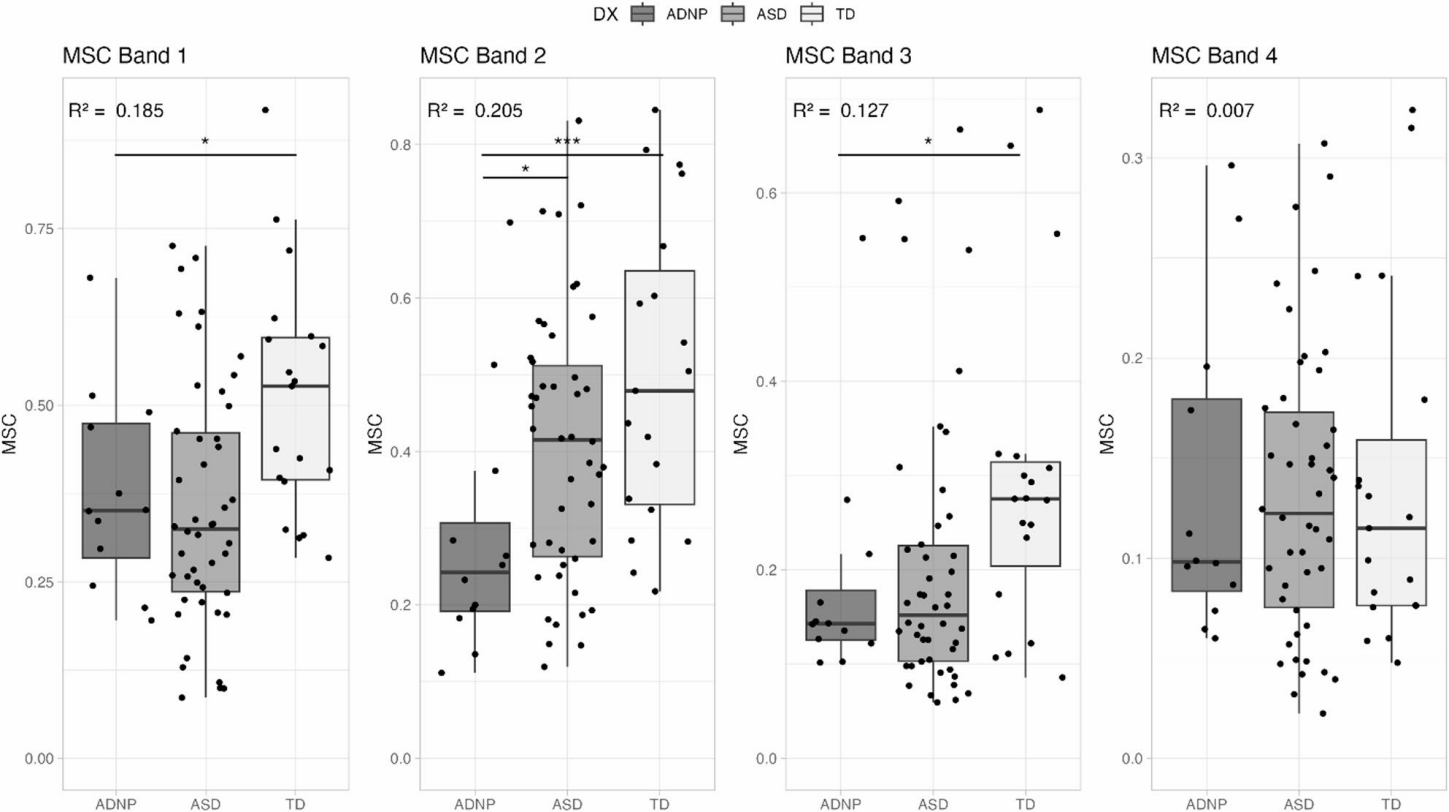
MSC Bands. Legend: Boxplots showing MSC Band 1 (6–12 Hz), Band 2 (14–28 Hz), Band 3 (30–40 Hz), and Band 4 (42–48 Hz). Colors represent the different groups: dark grey – ADNP, medium grey – ASD, and light grey – TD. Asterisks represent significance levels: * <0.05, ** <0.01, ***<0.001; *p* values reflect group differences from the regression model. R^2^ value located in the top left-hand corner

MSC Band 1 (6–12 Hz) was significantly weaker in the ADNP group compared to the TD group, but not compared to the ASD group (Table 2; Fig. 4). MSC Band 2 (14–28 Hz) was significantly weaker in the ADNP group compared to both TD and ASD groups. MSC Band 3 (30–40 Hz) was significantly weaker in the ADNP group compared to the TD group and no differences were identified among groups for MSC Band 4 (42–48 Hz).

## Discussion

This study used tVEPs to examine excitatory and inhibitory neurotransmission in a group of children with ADNP syndrome. Significant differences in tVEP responses were identified in the ADNP group compared to both the ASD and TD groups. Specifically, P_60_-N_75_ amplitude, which reflects initial afferent input to primary visual cortex, was significantly smaller in the ADNP group compared to the TD group, reflecting weaker postsynaptic excitatory activity. A subsequent loss of inhibitory postsynaptic activity, evidenced by smaller N_75_-P_100_ amplitude was also identified in the ADNP group compared to the TD group. This loss might simply reflect the weaker excitatory input to the cortex. Trends in these data indicate the ADNP group had even smaller responses than the ASD group. The ASD group consistently fell in between the ADNP and TD groups, which likely reflects the heterogeneity in ASD phenotype. The patterns observed here are similar to prior studies showing smaller responses in autistic children compared to controls and, on average, even smaller responses in those with specific rare genetic syndromes associated with autism [14, 18, 19]. Results from other genetic neurodevelopmental disorders have shown reduced N_75_-P_100_ amplitudes in children with PMS, Rett syndrome, CDKL5 deficiency disorder, and MECP2 duplication syndrome compared to a TD group, and P_100_-N_135_ in children with Rett and MECP2 duplication compared to a TD group [14, 16].

Response latencies were longer in the ADNP group compared to both the ASD and TD groups for P_60_ and N_75_. P_60_ latencies specifically indicate a delay in information being transmitted to the visual cortex. Interestingly, prior research has identified no difference in latency between ASD and TD groups [18], which is supported by these data as well. Studies in other developmental disorders including Rett and Fragile X syndromes similarly have not found a difference in VEP latency variables [15, 17]. Similarly, a study investigating Rett syndrome, CDKL5 deficiency disorder, MECP2 duplication syndrome, and FOXG1 syndrome did not find differences in latency for any disorders compared to a TD group [16]. The longer latencies identified here may therefore be a finding specific to ADNP syndrome and warrant further study in larger samples.

MSC bands measure relative signal power and adjust for inter-individual differences in amplitude. Higher MSC values indicate greater signal to noise. Children with ADNP syndrome had weaker responses compared to the TD group in Bands 1, 2, and 3, with the difference in Band 2 being the most striking. Children with ADNP syndrome showed weaker responses in Band 2 compared to both the TD and ASD groups. Band 2 reflects activity in the beta range (14–28 Hz) and is strongly correlated with the integrity of the waveform. Weaker responses here suggest reduced processing of visual stimuli in mid-range frequencies. Reduced Band 1 activity reflects activity in the theta and alpha range (6–12 Hz), and Bands 3 and 4 represent activity in the gamma range (30–40 Hz, 42–48 Hz, respectively). Results from prior research have shown a reduction of MSC and signal power in Bands 2, 3, and 4 in those with ASD compared to their siblings and TD controls [18, 19]. Similarly, a study in PMS showed reduced MSC (relative power) in Bands 2, 3, and 4 in children with PMS compared to their siblings and TD controls [14] and there may be shared mechanisms underlying PMS and ADNP syndromes [20]. Overall, there appears to be deficits relative to controls across the entire frequency range.

Findings from an open-label study evaluating low-dose ketamine in ADNP syndrome were promising with many clinician and caregiver rating scales showing improvement [21]. Exploratory EEG data in response to ketamine showed an increase in gamma band activity in response to 40 Hz stimulation, suggesting enhanced glutamatergic functioning. This may indicate ADNP syndrome is associated with lower levels of excitatory cortical activity, which if targeted by an intervention, may result in improvement of symptoms. This hypothesis is supported by the data in this study, which showed reduced P_60_- N_75_ deflections. The finding of prominent, albeit diminished, N_75_-P_100_ amplitudes may reflect intact cortical inhibition elicited by weakened cortical input. Further exploration in larger samples is necessary, particularly the ADNP group, as some variables had residuals that deviated from normality due to the small sample size.

Limitations of this study include an uneven male: female ratio between groups and a small sample size, especially for the ADNP group. In addition, this study included a wide age range of participants and, while age was controlled for, additional studies in a limited age range may be helpful. Replication with a larger ADNP cohort is indicated to further assess VEP differences identified here and to facilitate investigation of genotype-phenotype associations. Exploring relationships between VEP findings and both genotype and clinical outcome assessments are important future directions. Specifically, larger samples will allow for the examination of possible VEP relationships with cognitive/developmental level, ASD status, and sensory reactivity differences in individuals with ADNP syndrome.

## Conclusions

Overall, this study identifies abnormalities in tVEP responses in children with ADNP syndrome. Similar to previous studies in autism and associated genetic syndromes, amplitudes were weaker in the ADNP group compared to controls. In contrast to prior studies, longer latencies in the ADNP group represent findings that may be specific to ADNP syndrome and indicate an important area for future work. Continued exploration of VEPs in ADNP is warranted to further uncover possible electrophysiological biomarkers of this syndrome for use in clinical trials and to better understand underlying neuropathophysiology, genotype-phenotype relationships and treatment targets.

## Data Availability

The datasets used and/or analyzed during the current study are available from the corresponding author on reasonable request.

## Abbreviations

ADNP: Activity dependent neuroprotective protein
ADOS-2: Autism Diagnostic Observation Schedule – 2nd Edition
ASD: Autism spectrum disorder
DSM-5: Diagnostic and statistical manual of mental disorders, 5th edition
EEG: Electroencephalogram
IQ: Intellectual quotient
MSC: Magnitude-squared coherence
PMS: Phelan-McDermid syndrome
TD: Typically developing
tVEP: Transient visual evoked potential
VEP: Visual evoked potential

## References

[1] D’Incal CP, Van Rossem KE, De Man K, Konings A, Van Dijck A, Rizzuti L, et al. Chromatin remodeler Activity-Dependent Neuroprotective Protein (ADNP) contributes to syndromic autism. Clin Epigenetics. 2023;15(1):45. 36945042 10.1186/s13148-023-01450-8 PMC10031977

[2] Arnett AB, Rhoads CL, Hoekzema K, Turner TN, Gerdts J, Wallace AS, et al. The autism spectrum phenotype in ADNP syndrome. Autism Res. 2018;11(9):1300–10. 30107084 10.1002/aur.1980 PMC6203613

[3] Gozes I, Van Dijck A, Hacohen-Kleiman G, Grigg I, Karmon G, Giladi E, et al. Premature primary tooth eruption in cognitive/motor-delayed ADNP-mutated children. Transl Psychiatry. 2017;7(2):e1043. 28221363 10.1038/tp.2017.27 PMC5438031

[4] Pascolini G, Agolini E, Majore S, Novelli A, Grammatico P, Digilio MC. Helsmoortel-Van der Aa Syndrome as emerging clinical diagnosis in intellectually disabled children with autistic traits and ocular involvement. Eur J Paediatr Neurol. 2018;22(3):552–7. 29475819 10.1016/j.ejpn.2018.01.024

[5] Pascolini G, Di Zenzo G, Panebianco A, Didona B, Gozes I. Extended phenotypic characterization of a novel Helsmoortel-van der Aa syndrome case series. Am J Med Genet A. 2024;194(5):e63539. 38204290 10.1002/ajmg.a.63539

[6] Szabó TM, Balogh I, Ujfalusi A, Szűcs Z, Madar L, Koczok K, et al. Helsmoortel–Van der Aa syndrome—cardiothoracic and ectodermal manifestations in two patients as further support of a previous observation on phenotypic overlap with RASopathies. Genes. 2022;13(12):2367. 10.3390/genes13122367. 10.3390/genes13122367 PMC9778517 36553633

[7] Van Dijck A, Vandeweyer G, Kooy F. ADNP-Related Disorder. In: Adam MP, Feldman J, Mirzaa GM, Pagon RA, Wallace SE, Amemiya A, editors. GeneReviews(^®^). Seattle (WA): University of Washington, Seattle Copyright © 1993–2025, University of Washington, Seattle. GeneReviews is a registered trademark of the University of Washington, Seattle. All rights reserved.; 1993.

[8] Van Dijck A, Vulto-van Silfhout AT, Cappuyns E, van der Werf IM, Mancini GM, Tzschach A, et al. Clinical Presentation of a Complex Neurodevelopmental Disorder Caused by Mutations in ADNP. Biol Psychiatry. 2019;85(4):287–97. 29724491 10.1016/j.biopsych.2018.02.1173 PMC6139063

[9] Siper PM, Layton C, Levy T, Lurie S, Benrey N, Zweifach J, et al. Sensory reactivity symptoms are a core feature of ADNP syndrome irrespective of autism diagnosis. Genes (Basel). 2021;12(3):351. 10.3390/genes12030351 PMC7997330 33673501

[10] Fastman J, Kolevzon A. ADNP Syndrome: A qualitative assessment of symptoms, therapies, and challenges. Child (Basel). 2023;10(3):593. 10.3390/children10030593 PMC10047312 36980151

[11] Karmon G, Sragovich S, Hacohen-Kleiman G, Ben-Horin-Hazak I, Kasparek P, Schuster B, et al. Novel ADNP Syndrome Mice Reveal Dramatic Sex-Specific Peripheral Gene Expression With Brain Synaptic and Tau Pathologies. Biol Psychiatry. 2022;92(1):81–95. 34865853 10.1016/j.biopsych.2021.09.018

[12] Odom JV, Bach M, Brigell M, Holder GE, McCulloch DL, Mizota A, Tormene AP,International Society for Clinical Electrophysiology of Vision. ISCEV standard for clinical visual evoked potentials: (2016 update). Documenta ophthalmologica. Advances in ophthalmology. 2016;133(1):1–9. 10.1007/s10633-016-9553-y. 10.1007/s10633-016-9553-y 27443562

[13] Zemon V, Gordon J. Luminance-contrast mechanisms in humans: visual evoked potentials and anonlinear model. Vision research. 2006;46(24):4163–80. 10.1016/j.visres.2006.07.007. 10.1016/j.visres.2006.07.007 16997347

[14] Siper PM, Rowe MA, Guillory SB, Rouhandeh AA, George-Jones JL, Tavassoli T, et al. Visual Evoked Potential Abnormalities in Phelan-McDermid Syndrome. J Am Acad Child Adolesc Psychiatry. 2022;61(4):565–e741. 34303785 10.1016/j.jaac.2021.07.006 PMC8782912

[15] LeBlanc JJ, DeGregorio G, Centofante E, Vogel-Farley VK, Barnes K, Kaufmann WE, et al. Visual evoked potentials detect cortical processing deficits in Rett syndrome. Ann Neurol. 2015;78(5):775–86. 26332183 10.1002/ana.24513 PMC7374762

[16] Saby JN, Peters SU, Benke TA, Standridge SM, Swanson LC, Lieberman DN, et al. Comparison of evoked potentials across four related developmental encephalopathies. J Neurodevelopmental Disorders. 2023;15(1):10. 10.1186/s11689-023-09479-9 PMC9985257 36870948

[17] Knoth IS, Vannasing P, Major P, Michaud JL, Lippé S. Alterations of visual and auditory evoked potentials in fragile X syndrome. Int J Dev Neurosci. 2014;36:90–7. 24875778 10.1016/j.ijdevneu.2014.05.003

[18] Siper PM, Zemon V, Gordon J, George-Jones J, Lurie S, Zweifach J, et al. Rapid and Objective Assessment of Neural Function in Autism Spectrum Disorder Using Transient Visual Evoked Potentials. PLoS ONE. 2016;11(10):e0164422. 27716799 10.1371/journal.pone.0164422 PMC5055293

[19] Brittenham C, Gordon J, Zemon VM, Siper PM. Objective frequency analysis of transient visual evoked potentials in autistic children. Autism Res. 2022;15(3):464–80. 34908250 10.1002/aur.2654

[20] Ivashko-Pachima Y, Ganaiem M, Ben-Horin-Hazak I, Lobyntseva A, Bellaiche N, Fischer I, et al. SH3- and actin-binding domains connect ADNP and SHANK3, revealing a fundamental shared mechanism underlying autism. Mol Psychiatry. 2022;27(8):3316–27. 35538192 10.1038/s41380-022-01603-w

[21] Kolevzon A, Levy T, Barkley S, Bedrosian-Sermone S, Davis M, Foss-Feig J, et al. An open-label study evaluating the safety, behavioral, and electrophysiological outcomes of low-dose ketamine in children with ADNP syndrome. Hum Genet Genomics Adv. 2022;3(4):100138. 10.1016/j.xhgg.2022.100138 PMC9471202 36119806

